# Codon Usage in the *Iflaviridae* Family Is Not Diverse Though the Family Members Are Isolated from Diverse Host Taxa

**DOI:** 10.3390/v11121087

**Published:** 2019-11-22

**Authors:** Sheng-Lin Shi, Run-Xi Xia

**Affiliations:** College of Bioscience and Biotechnology, Shenyang Agricultural University, Shenyang 110866, China; shishenglin@126.com

**Keywords:** Iflavirus, Nucleotide composition, Codon bias, Preferred codons, Selection pressure

## Abstract

All iflavirus members belong to the unique genus, *Iflavirus*, of the family, *Iflaviridae*. The host taxa and sequence identities of these viruses are diverse. A codon usage bias, maintained by a balance between selection, mutation, and genetic drift, exists in a wide variety of organisms. We characterized the codon usage patterns of 44 iflavirus genomes that were isolated from the classes, Insecta, Arachnida, Mammalia, and Malacostraca. Iflaviruses lack a strong codon usage bias when they are evaluated using an effective number of codons. The odds ratios of the majority of dinucleotides are within the normal range. However, the dinucleotides at the 1st–2nd codon positions are more biased than those at the 2nd–3rd codon positions. Plots of effective numbers of codons, relative neutrality analysis, and PR2 bias analysis all indicate that selection pressure dominates mutations in shaping codon usage patterns in the family, *Iflaviridae*. When these viruses were grouped into their host taxa, we found that the indices, including the nucleotide composition, effective number of codons, relative synonymous codon usage, and the influencing factors behind the codon usage patterns, all show that there are non-significant differences between the six host-taxa-groups. Our results disagree with our assumption that diverse viruses should possess diverse codon usage patterns, suggesting that the nucleotide composition and codon usage in the family, *Iflaviridae*, are not host taxa-specific signatures.

## 1. Introduction

Synonymous codon usage biases (codon usage biases or codon biases) are widespread and exist in a number of organisms, from viruses to mammals. Such a bias refers to the symptom of a given organism preferring a particular codon, and it is central to fields from molecular evolution to biotechnology [1]. Codon usage affects protein biogenesis, including translation efficiency and gene function, beyond specifying the protein sequence [2,3]. Synonymous mutations can interrupt the formation of correct mRNA secondary structures, alter the start of transcription, and thus may not be functionally neutral [4]. Moreover, a recent research found that the codon usage of the GFP gene influences bacterial fitness through RNA toxicity [5] and thus highlights the importance of research on codon usage. Understanding the extent and causes of codon usage biases is essential to research on viral evolution and transmission, and it is also important for interpreting virus–host interplay [6]. A balance between selection, mutation, and genetic drift maintains the codon usage bias. For instance, codon usage patterns in the families, *Flaviviridae* and *Parvoviridae*, are affected mainly by natural selection [7,8]. However, there is no common rule as to the main factor influencing codon biases. In other words, it is hard to predict the relative roles of selection and mutations in dominating the codon usage in a given organism, without a detailed analysis.

Through the metagenomics of the 21st century, more and more novel RNA virus species were discovered. According to the International Committee on Taxonomy of Viruses (ICTV), members of the *Iflaviridae* family are together classified into the single genus, *Iflavirus* [9]. Most iflaviruses have been isolated from arthropods, primarily insects in the orders of *Lepidoptera*, *Hymenoptera*, *Hemiptera*, *Diptera*, and so forth. The infections of iflaviruses can be symptomless or result in developmental abnormalities, behavior changes, and premature mortality, and hence, they usually cause great economic losses in sericulture and apiculture [10,11,12]. Additionally, iflaviruses in the insect population that are used for baculovirus insecticide production are problematic, because they diminish the growth of insects and reduce the production of occlusion bodies [13]. Iflaviruses possess a single copy of a single stranded RNA genome, which encodes a single polyprotein which has a conserved genome organization. Structural proteins (VP1, VP2, VP3, and VP4) are located at the N-terminus, and non-structural proteins (RNA helicase, protease, and RNA-dependent RNA polymerase) are located at the C-terminus. Translation initiates at an internal ribosome entry site in the 5′ untranslated region and a poly (A) tail follows the 3′ untranslated region [9].

The *Iflaviridae* family has grown rapidly due to the development of next-generation sequencing technology. Work on these emerging iflaviruses offers a unique and exciting opportunity for researchers, though the task of studying the RNA viruses of insects comes with particular challenges [14]. Iflaviruses are diverse in their sequence identities and host taxa. For example, the polyprotein sequence of *Helicoverpa armigera* iflavirus shows a relatively higher similarity (61.0%) with the protein of the *Lymantria dispar* Iflavirus 1 [15]. However, the two mulberry silkworm infecting iflaviruses, *Bombyx mori* iflavirus and infectious flacherie virus, show only a relatively lower identity (23%) [16]. Furthermore, iflaviruses do not follow the same evolutionary path as their hosts at the order level, because they do not form a single clade, according to the insect-infected order [17]. So far, the host range of most iflaviruses has not been examined beyond the original host descriptions. However, at least 64 host species, spanning eight orders, have been found being infected with the deformed wing virus [18].

Presently, few studies have focused on the evolution of viruses of the *Iflaviridae* family, and the codon usage patterns in iflaviruses remain unknown. Different viruses differ in their natural hosts, and different hosts differ in their tRNA pools. Viruses rely on the tRNAs of their host for protein synthesis and thus coevolve with their host to efficiently use host resources [19]. We assumed that similar viruses possess similar codon usage patterns, and diverse viruses diverge in their codon usage patterns too. Iflaviruses are diverse in their sequences and host taxa, and hence their codon usage patterns may diverge. To verify this assumption, we used 44 iflavirus genomes to infer the codon usage patterns in the *Iflaviridae* family. We found that the nucleotide composition and codon usage patterns in the *Iflaviridae* family are relatively uniform and not as diverse as we expected.

## 2. Materials and Methods

### 2.1. Genome Sequences

In the GenBank genome database (https://www.ncbi.nlm.nih.gov/genome/), a total of 42 virus species of the *Iflaviridae* family were available on May 6, 2019. Though 27 of the 42 virus species were not listed in the current ICTV Online (10th) Report (http://ictv.global/report) and were placed under an “unclassified” node of *Iflaviridae* family in the NCBI viral taxonomy database, we downloaded the genomes of all these viruses in our analysis for the following reasons: (1) sequences in GenBank genome database are reference sequences and are reliable [20], and (2) sequence data provide abundant information for taxonomy and viruses identified from sequence data are bona fide viruses [21]. There are 44 iflavirus reference genomes because three strains of *Culex Iflavi-like virus 4* were included (Table S1). We extracted the coding region of the polyprotein in each genome, deleted the codons containing ambiguous bases, and merged the two truncating coding regions in the *Nesidiocoris tenuis* iflavirus 1 genome to form an artificial single coding region to facilitate our analysis.

### 2.2. Nucleotide Composition

We calculated the base compositions of synonymous codons for each genome, including the overall base compositions of guanine (G), cytosine (C), adenine (A), and thymine (T) [termed Gs, Cs, As, and Ts, respectively], and the base compositions of G, C, A, and T at the third codon position (termed G3s, C3s, A3s, and T3s, respectively). We calculated the base compositions of guanine plus cytosine (GC) at the first, second and third positions of synonymous codons (termed GC1s, GC2s and GC3s, respectively). For each genome, we also calculated the dinucleotide compositions at the synonymous codon’s 1st–2nd positions and at the synonymous codon’s 2nd–3rd positions. The dinucleotide odds ratio was calculated, following the method described by Karlin and Burge [22]. The odds ratio *ρ*_xy_ = *f*_xy_/*f*_x_*f*_y_, where *f*_x_ and *f*_y_ denote the frequency of nucleotides, *X* and *Y*, respectively, *f*_x_*f*_y_ denotes the expected frequency of dinucleotide *XY*, and *f*_xy_ denotes the observed frequency of dinucleotide *XY*. As a conservative criterion, for *ρ*_xy_ ≥ 1.23 or ≤ 0.78, the *XY* pair is considered to be over- or under-represented; for 1.20 ≤ *ρ*_xy_< 1.23 or 0.79 ≤ *ρ*_xy_< 0.82, the *XY* pair is considered to be marginally high or marginally low; and for 0.82 ≤ *ρ*_xy_ ≤ 1.19, the *XY* pair is considered to be within the normal range.

### 2.3. Relative Synonymous Codon Usage

The relative synonymous codon usage (RSCU) is defined as a codon’s observed frequency, divided by its expected frequency, under the assumption of an equal usage of all codons for an amino acid [23]. A codon with an RSCU value greater than one represents a positive codon usage bias, while a codon with an RSCU value less than one represents a negative codon usage bias. For RSCU < 0.6 or > 1.6, the codon is considered to be under- or over-represented. We simplified the previously reported method [24] to identify preferred codons. For each amino acid, a Chi-Square goodness-of-fit test was performed to examine the differences between the codons’ frequencies. When the *p* value < 0.05, we referred to the codon with the highest RSCU value as the preferred codon. When the *p* value > 0.05, the amino acid adopted codons equally, and no preferred codon was identified. Two preferred codons were referred to for the six-codon amino acids (Arg, Leu, Ser) due to the higher RSCU values of these codons in our analysis.

The cosine similarity index *R(A,B)* was proposed previously to estimate the degree of similarity between a virus and its specific host with respect to the aspect of the overall codon usage patterns [25]. Here, we applied this index to compute the pairwise similarities between viruses of different host taxa.

### 2.4. Effective Number of Codons and ENc-Plot

The effective number of codons (ENc value) is a parameter, defined as the number of equally used codons that would yield the observed level of codon usage, and it is thus widely used to measure the degree of codon usage bias [26]. If each amino acid used only one codon, the ENc value would be 20 (extreme codon bias), and if all amino acids used all codons equally, the ENc value would be 61 (no codon bias). In general, a codon’s bias is described as strong when its ENc value is not greater than 35, described as weak when its ENc value is greater than 45, and described as moderate when its ENc value is between these values [27,28].

The ENc-plot analysis plots an ENc value against the GC3s content to examine the influence of nucleotide composition on codon usage. The expected ENc value assumes no selection and GC bias at silent sites due to mutation, or no selection and no GC mutation bias, and can be calculated from the GC3s content, according to the equation provided by Wright [26]. If a gene is subject to GC compositional constraints, it will lie on or just below the curve of the predicted ENc values. However, such a gene may either be subject to a GC-biased mutation pressure or under selection (negative/positive) for codons ending in G and/or C.

### 2.5. Relative Neutrality Plot and Parity Rule 2 Bias Plot

The relative neutrality plot analysis estimates the extent of the neutrality of the directional mutation pressure against selection [29]. In the analysis, *P*_1_, *P*_2_, and *P*_3_ are the observed GC contents of the first, second, and third codon positions of an individual gene (*P*_12_ is the average of *P*_1_ and *P*_2_), and eight codons (ATG, TGG, ATA, ATT, ATC, TAA, TAG, or TGA) are excluded from the calculation. When plotting *P*_12_ against *P*_3_, the regression coefficient is the mutation-selection equilibrium coefficient.

The parity rule 2 (PR2) refers to the base composition at equilibrium within one strand of DNA. At equilibrium, the frequencies of the four bases under non-strand bias conditions follows A = T and G = C within each strand [30]. In the PR2 bias plot, the G/(G + C) value is plotted as abscissa, and the A/(A + T) value is plotted as ordinate [31]. The center of the plot, where both coordinates are 0.5, represents the point where the nucleotide composition follows the PR2 rule, and the distance and direction from the center represent the extent and direction of the bias from the PR2 rule. We plotted the bases at the third codon position of the four-codon amino acids, because this type of plot is more informative. The four-codon amino acids are alanine, arginine (CGA, CGT, CGG, and CGC), glycine, leucine (CTA, CTT, CTG, and CTC), proline, serine (TCA, TCT, TCG, and TCC), threonine, and valine.

### 2.6. Correspondence Analysis

Correspondence analysis is widely used to evaluate the major variation trend in codon usage among coding sequences [32]. In the correspondence analysis, each coding sequence is represented as a 59-dimensional vector, and each dimension corresponds to the RSCU value of one sense codon (excluding AUG, UGG and three stop codons). The correspondence analysis partitions the variation along 59 orthogonal axes, and the first two axes often explain the largest fractions of variation in the data.

### 2.7. Software and Calculation

We used the Perl script to calculate the nucleotide and dinucleotide compositions, as well as the *P*_1_, *P*_2_, *P*_3_ values. We used the CodonW software to calculate the ENc and RSCU values and to perform the correspondence analysis. The statistical analysis and similarity index calculation were performed using the SPSS 16.0 software. The heat map was drawn using the HemI software, and the row and column data of the heat map were clustered by the Average Linkage method using the Euclidean distance [33].

## 3. Results

### 3.1. Nucleotide Composition

The nucleotide compositions (Table S1) were biased in iflaviruses. Within each genome, the frequencies of Gs, Cs, As, and Ts differed significantly, and the frequencies of G3s, C3s, A3s, and T3s also differed significantly (*p* < 0.001, goodness-of-fit test). Furthermore, the ratio of Gs/Cs/As/Ts differed significantly from the ratio of G3s/C3s/A3s/T3s in each genome (*p* < 0.001, Chi-square test). The standard deviation (SD) was used to quantify the variation across the four bases of a genome. The SD values of the overall base compositions (Gs, Cs, As and Ts) ranged from 2.73% (*Lygus lineolaris* virus 1) to 10.16% (*Opsiphanes invirae* iflavirus 1), while the SD values of the base compositions at the third codon position (G3s, C3s, A3s and T3s) ranged from 3.81% (Diamondback moth iflavirus) to 22.14% (*Helicoverpa armigera* iflavirus). This result indicated that, in a genome, the base compositions at the third codon position were more diverse than the overall base compositions.

All the 44 iflaviruses were grouped, according to their host taxa, into Diptera viruses, Hemiptera viruses, Hymenoptera viruses, Lepidoptera viruses, Arachnida viruses and non-Insect viruses (Malacostraca viruses and Mammalia viruses). This time, no significant differences of the aforementioned nucleotide compositions (Gs, Cs, As, Ts, G3s, C3s, A3s, and T3s, respectively) were detected between the host-taxa-groups (Kruskal–Wallis Test, Benjamini–Hochberg correction, with a false discovery rate of q* = 0.05, was used to set the significance level), suggesting that nucleotide contents have no direct association with host taxa. When we calculated the means for the four bases of all viruses, the ratios of Gs/Cs/As/Ts and G3s/C3s/A3s/T3s were 21/18/30/31 and 15/13/29/43, respectively. This result indicated that iflaviruses tended to use A/T bases, especially at the third codon position, and selection might exist to shape the nucleotide composition.

### 3.2. Dinucleotide Composition

**Figure 1:**
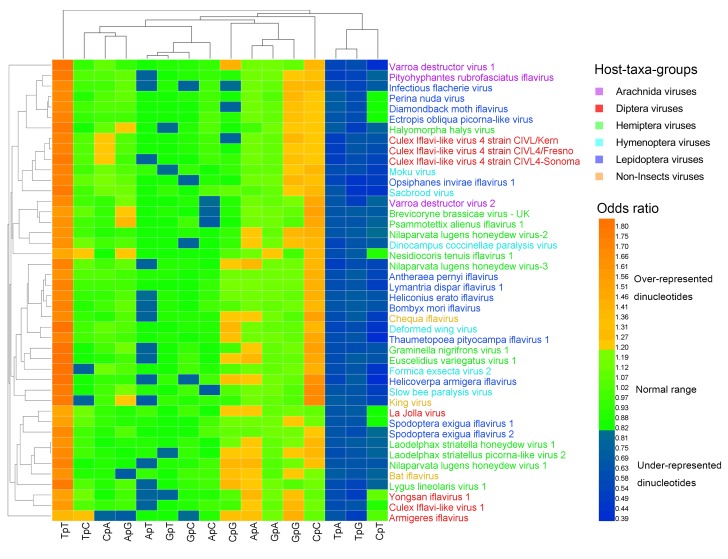
Heat map of the dinucleotide composition at the 1st–2nd codon positions. Iflavirus genomes were clustered independently of viral host taxa, implying that there exist other factors behind dinucleotide composition. The viruses and dinucleotides were clustered by the Average Linkage method using the Euclidean distance.

**Figure 2:**
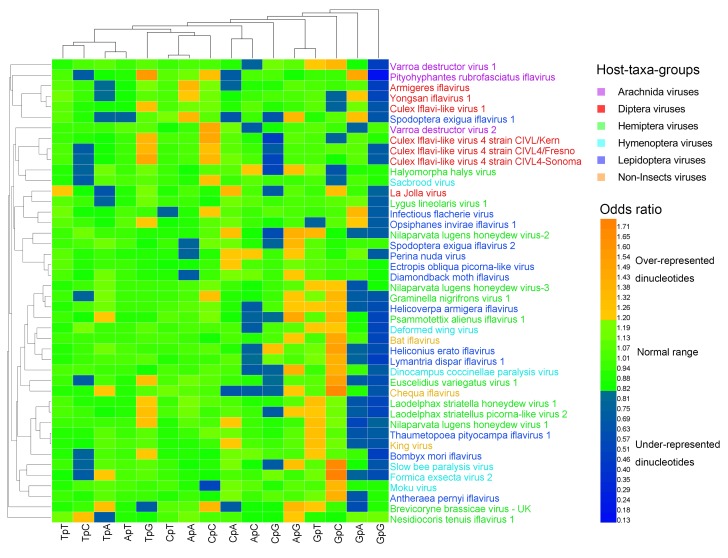
Heat map of the dinucleotide composition at the 2nd–3rd codon positions. Iflavirus genomes were clustered independently of viral host taxa, implying that there exist other factors behind dinucleotide composition. The viruses and dinucleotides were clustered by the Average Linkage method using the Euclidean distance.

Measured by the odds ratio (*ρ*_xy_), the majority of dinucleotides were within the normal range (0.82 ≤ *ρ*_xy_ ≤ 1.19). The over-represented (or marginally high) dinucleotides (*ρ*_xy_ ≥ 1.20) and the under-represented (or marginally low) dinucleotides (*ρ*_xy_ < 0.82) were the minority (Table S2, Figure 1, and Figure 2). At the 1st–2nd codon positions, 20% (141/704) of dinucleotides were over-represented (or marginally high), 23% (159/704) of dinucleotides were under-represented (or marginally low), and 57% (404/704) of dinucleotides were within the normal range. The most over-represented (or marginally high) dinucleotides included TpT (in 44/44 genomes; 100%) and CpC (in 37/44 genomes; 84%), and the most under-represented (or marginally low) dinucleotides included TpA (in 44/44 genomes; 100%), TpG (in 44/44 genomes; 100%), and CpT (in 35/44 genomes; 80%). At the 2nd–3rd codon positions, 13% (88/704) of dinucleotides were over-represented (or marginally high), 15% (105/704) of dinucleotides were under-represented (or marginally low), and 73% (511/704) of dinucleotides were within the normal range. The most over-represented (or marginally high) dinucleotide was GpC (in 17/44 genomes; 39%), and the most under-represented (or marginally low) dinucleotides included GpG (in 32/44 genomes; 73%) and GpA (in 16/44 genomes; 36%).

The heat map of the dinucleotides at the 1st–2nd codon positions (Figure 1) failed to cluster the 44 viral genomes in accordance with their host taxa, though certain viruses from the same host taxa were clustered together. The three non-Insect viral genomes and the six Hymenoptera viral genomes were all scattered randomly with the other viral genomes, indicating that the dinucleotide bias was independent of the viral host taxa. The heat map of the dinucleotides at the 2nd–3rd codon positions (Figure 2) failed to cluster the 44 viral genomes in accordance with their host taxa too, but it showed a less significant dinucleotide bias.

### 3.3. Synonymous Codon Usage

Codon usage patterns in iflaviruses were investigated by calculating the codon frequencies and RSCU values. For the six host-taxa-groups, only the lysine (Lys) in the non-Insect group was encoded randomly (*p* = 0.380, goodness-of-fit test). We identified the preferred codons for the amino acids that were encoded unequally in the six host-taxa-groups, according to their RSCU values (Table S3, Table 1). Two preferred codons were identified for each six-codon amino acid, and one preferred codon was identified for the other amino acids. The result showed that only three preferred codons (GGA for Gly, CTT for Leu, and TCA for Ser) in the Diptera viral group were outliers, as they were not identical to any preferred codons in other host-taxa-groups. Consequently, a total of 17 preferred codons were shared by all of the six host-taxa-groups, and four preferred codons were shared by five host-taxa-groups. The wobble positions of the preferred codons in all genomes were highly biased. Most shared preferred codons were strongly biased toward the T base in the third positions (15 out of 21 preferred codons). Conversely, only five codons ended with the A base, and one codon ended with the G base. Furthermore, the three outliers selected the A base twice and the T base once at their wobble positions.

**Figure 3:**
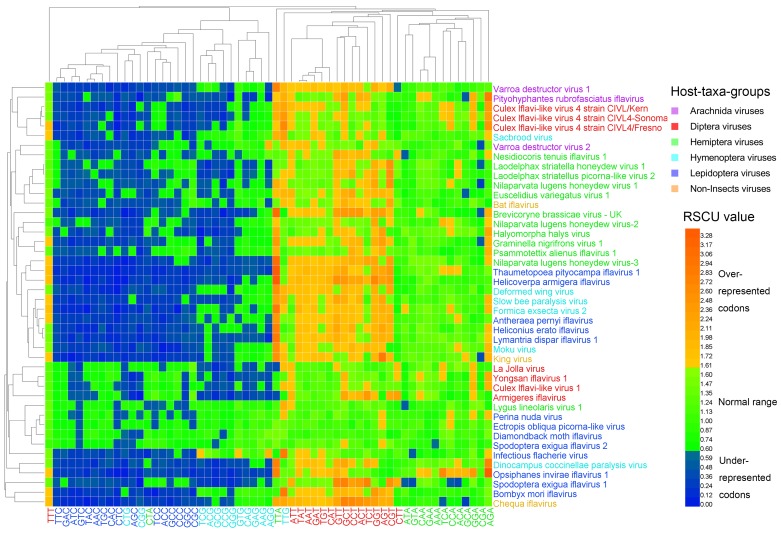
Heat map of the relative synonymous codon usage (RSCU). Iflavirus genomes were clustered in accordance partially with the viral host taxa, and codons were clustered in accordance mainly with the third base, indicating that viral host taxa impact codon usage, and the codon usage is highly biased toward T-ending codons. The viruses and codons were clustered by the Average Linkage method using the Euclidean distance.

The heat map of the RSCU values (Figure 3) clustered the 44 viral genomes only in accordance partially with their host taxa, in a similar way to the heat map of dinucleotides (Figure 1 and Figure 2). Codons that ended with G/C, except TTG, were clustered together, and codons that ended with A/T, except CTA, were clustered together. Thymine-ending codons in most iflaviruses were over-represented, A-ending codons (except CTA) in most iflaviruses were normal codons, and G/C-ending codons (except TTG) in most iflaviruses were under-represented. This result suggests that the *Iflaviridae* family members preferred A/T-ending codons over G/C-ending codons. Using the cosine similarity index, we measured the pairwise relationship across the six host-taxa-groups and found that the similarity indexes all scored high (ranging from 0.958 to 0.993), indicating a relatively closer relationship between these host-taxa-groups.

### 3.4. ENc Value and ENc-Plot Analysis

ENc values of the 44 viral genomes ranged from 37.47 (*Helicoverpa armigera* iflavirus) to 57.73 (Diamondback moth iflavirus), with a mean of 46.99 ± 5.02 (Table S1). The codon usage is regarded as strongly biased when the mean ENc value is lower than 35. Therefore, all of the 44 viral genomes lacked a strong codon bias. The ENc values of 16 genomes ranged from 37.47 to 44.91, and hence, these genomes possessed a moderate codon bias. The remaining 28 genomes possessed a weak codon bias. Among the six host-taxa-groups, the difference of ENc values was insignificant (*p* = 0.413, Kruskal Wallis Test), suggesting that the codon bias of iflaviruses is independent of viral host taxa.

**Figure 4:**
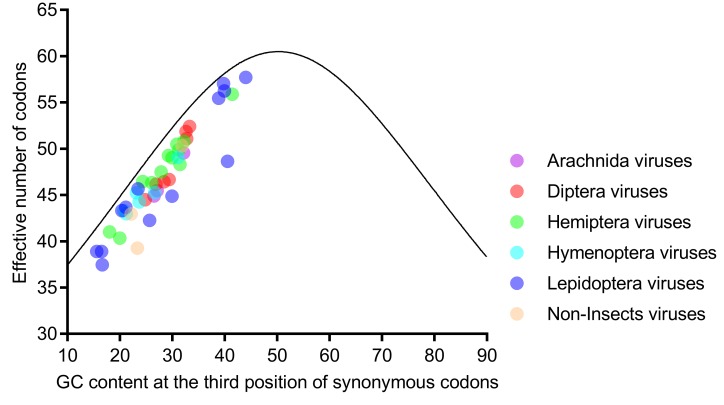
All genomes of the six host-taxa-groups interspersed and aligned along, but not on, the expected curve in the ENc (effective number of codons)-plot analysis, indicating that the factors influencing the codon usage in the six host-taxa-groups are similar, and mutation is not the main force. Each dot represents a viral genome corresponding to the two coordinate axes. The solid curve indicates the expected ENc values, assuming that the GC compositional constraints alone account for the codon usage.

Further analysis showed that the ENc values were strongly correlated with GC1s (*ρ* = 0.772, *p* < 0.001) and GC2s (*ρ* = 0.628, *p* < 0.001) and perfectly correlated with GC3s (*ρ* = 0.935, *p* < 0.001). Taken together with the result that all viral genomes were scattered along, but not on, the expected curve in the ENc-plot (Figure 4), we concluded that the nucleotide composition was not the only factor influencing codon usage, and selection might have an important role. Moreover, the ENc-plot separated the six host-taxa-groups unclearly, implying that the factors influencing the codon usage of the six host-taxa-groups are similar.

### 3.5. Relative Neutrality Plot and PR2 Bias Plot

**Figure 5:**
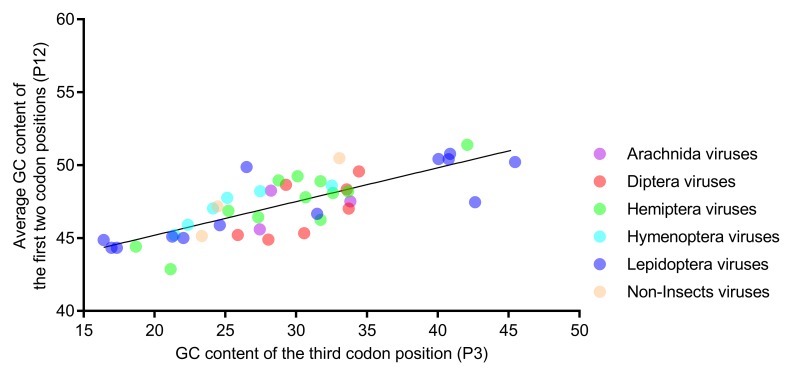
The regression slope of *P*_12_ (average of *P*_1_ and *P*_2_) against *P*_3_ was found to be low in the relative neutrality analysis, indicating that selection dominates mutations in shaping the nucleotide composition. Members of the six host-taxa-groups were all interspersed around the regression line, implying that the factors affecting the codon usage of different host-taxa-groups are similar. Each dot represents a viral genome corresponding to the two coordinate axes. The solid line indicates the regression, with equation Y = 0.231X + 40.57, R^2^ = 0.615, and *p* < 0.001.

Natural selection and mutational pressure are the two main factors that account for the codon usage variation in different organisms. The relative neutrality plot analysis (Figure 5) showed that the regression coefficient of *P*_12_ to *P*_3_ was 0.231 (*p* < 0.001), indicating a lower neutrality of *P*_12_, relative to *P*_3_. The results suggested that selection pressure dominated mutations in shaping the codon usage in iflavirus genomes. All of the dots were scattered randomly around the regression line, regardless of the viral host taxa, suggesting that the affecting factors are not host taxa-specific.

**Figure 6:**
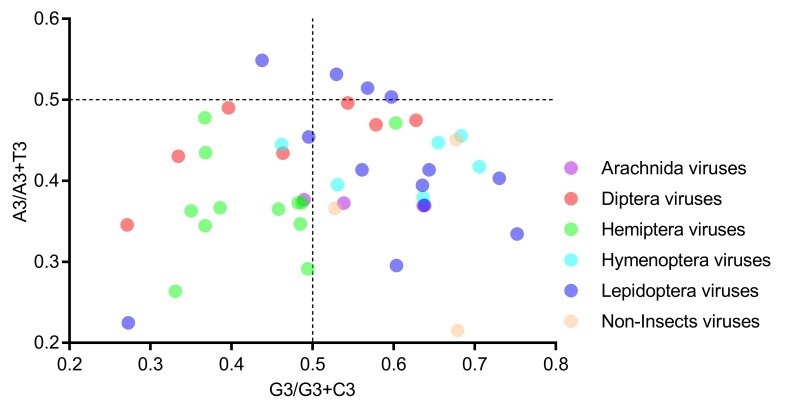
All viral genomes scattered away from the plot center of 0.5 in the PR2 bias analysis, suggesting that there exists a codon usage bias in iflavirus genomes. Members of the six host-taxa-groups were interspersed in the plot, implying that only an insignificant codon usage difference existed across viral genomes. Each dot represents a genome corresponding to the two coordinate axes. The two dashed lines indicate no GC bias or no AT bias, respectively. The plot center (both coordinates are 0.5) indicates a position without a base bias.

In the PR2 bias plot (Figure 6), no genome is located at the plot center (both coordinates are 0.5), and most genomes are located at the lower part, indicating a strong T bias at the third codon position. Moreover, neither the A/A+T bias nor the G/G+C bias can clearly separate the six host-taxa-groups, again suggesting that the codon usage bias in the *Iflaviridae* family is not directly related to the viral host taxa.

### 3.6. Correspondence Analysis

The first two axes in the correspondence analysis usually account for the major variation in the codon usage bias. In the present study, the first, second, third and fourth axes accounted for 31.59%, 19.20%, 12.60%, and 6.17% of the total variation, respectively. This result confirmed that the first two axes account for the major variation in iflavirus genomes.

**Figure 7:**
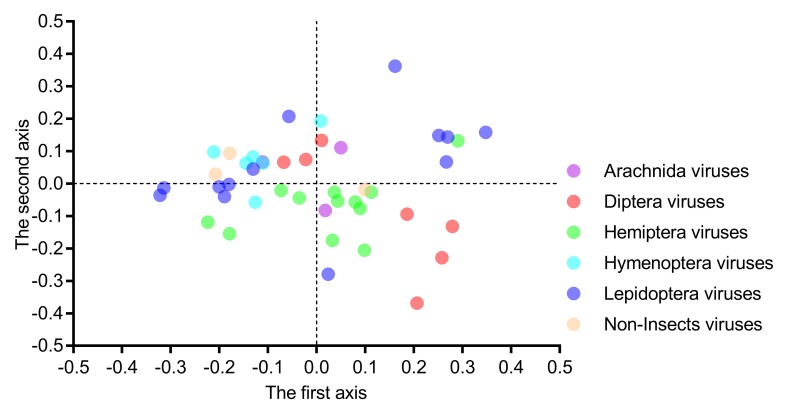
Both the first and second axes of the correspondence analysis failed to clearly separate the six viral group members, indicating that the factors influencing the codon usage in iflaviruses are independent of viral host taxa. Each dot represents a genome corresponding to the two coordinate axes. The two dashed lines indicate zero variations in the coordinates.

The correlation analysis indicated that the first axis was strongly correlated with GC1s (*ρ* = 0.799, *p* < 0.001) and GC2s (*ρ* = 0.662, *p* < 0.001) and perfectly correlated with GC3s (*ρ* = 0.938, *p* < 0.001) and ENc values (*ρ* = 0.922, *p* < 0.001). However, the second axis had no significant correlation with GC1s (*ρ* = 0.161, *p* = 0.493), GC2s (*ρ* = 0.067, *p* = 0.664), GC3s (*ρ* = 0.241, *p* = 0.116) and ENc values (*ρ* = 0.143, *p* = 0.354), confirming that the first axis represents the major factor shaping codon usage. These results, taken together, suggested that the nucleotide composition was not the only factor affecting the codon usage. When we plotted the 44 viral genomes on the first two main axes (Figure 7), the six scattered host-taxa-groups were independent of their host taxa too, and neither of the two axes could clearly separate these viruses. This result indicated that the factors affecting the codon choice of different host-taxa-groups in the *Iflaviridae* family were similar.

## 4. Discussion

Understanding the origins and evolution of RNA viruses is more difficult than obtaining the sequences of novel RNA viruses. A codon usage bias refers to an uneven use of synonymous codons. It is an important aspect of genome evolution and serves as a secondary genetic code guiding protein production [34]. We provide a detailed analysis of the nucleotide composition and codon usage in the *Iflaviridae* family. When evaluated in terms of their ENc value, the 44 viral genomes all lacked a strong codon bias. This result is consistent with the codon usage in most RNA viruses, such as the Citrus tristeza virus [35], Enterovirus [36], Hendra virus [37], and Nipah virus [38], in that they all lacked a strong codon bias. A possible explanation for the weak codon bias is that it is advantageous for efficient replication, re-adaption, and survival in host cells [39,40]. The similarity index *R(A,B)* has been widely used to represent the degree of similarity between a virus and its host, with respect to their overall codon usage patterns, and to further evaluate the potential role of the overall codon usage patterns of the host in the formation of the overall codon usage of a virus [25,38]. We used this index to infer the similarity between host-taxa-groups and found that all of the similarity values were at higher levels. We also found that iflaviruses from different host taxa shared most of the preferred codons, suggesting that the overall codon usage in iflaviruses is not diverse.

The usage of some codons, for example, AGG (Arg) and TTG (Leu), are unusual in prokaryotic and eukaryotic genomes [41,42]. In the *Iflaviridae* family, the usage of two codons, TTG (Leu) and CTA (Leu), were unusual too (Figure 3). The codon, TTG, unlike all of the other G/C-ending codons, was clustered with over-represented T-ending codons. Further, TTG was the only G-ending preferred codon and was shared by all the six host-taxa-groups. On the other hand, the codon, CTA, was clustered with the under-represented C-ending codons, though most A-ending codons were within the normal range or over-represented. The unusual usage of the two codons may be a consequence of the biased dinucleotide usage. The over- and under-represented TTG and CTA corresponded to the over- and under-represented dinucleotides, TpT and CpT, at the 1st–2nd codon positions, respectively. However, the selection of the ending base for the two codons cannot be explained by the dinucleotide usage bias at the 2nd–3rd codon positions (GpC is over-represented, and GpG and GpA are under-represented).

Detecting the main factors affecting the codon usage patterns is always the core of codon usage investigation. In the standard evolutionary theory, the forces accounting for adaptation and phenotypic evolution are mutation, genetic drift, recombination, and selection [43]. In viruses, natural selection and mutation pressure are two key maintaining factors, behind the codon usage patterns. In certain organisms, a mutational bias plays important roles in shaping codon usage, whereas natural selection is the main force in others [2]. In the present study, we found that natural selection dominated mutational pressure in shaping codon usage. This finding agrees with the results of other studies on the Citrus tristeza virus [35], Hendra virus [37] and Influenza C virus [44], in that natural selection plays a more important role in shaping the codon usage, but it disagrees with the results of other studies on the Ebola virus [39] and Enterovirus [36] in that mutational pressure is more important in shaping the codon usage.

Viruses are intracellular pathogens and dependent on their hosts’ tRNAs for translation. Consequently, viruses exploit and coevolve with their host to prosper in cellular environments. Analyzing the codon usage in viruses, along with their hosts, will be important to understand viral evolution. The codon usage patterns of certain viruses are influenced by their hosts [45,46], although differences in codon preferences exist between viruses and their hosts [36,39,40]. However, we found that the nucleotide composition and codon usage in the *Iflaviridae* family does not vary in accordance with viral host taxa. This result is consistent with the findings that an animal host has a relatively smaller impact on the viral dinucleotide composition [47]; moreover, a host association drives the viral codon usage, but a host does not serve as a template for the viral codon usage [48]. Virus host shift events and host range affect viral codon usage patterns in that narrow-host-range viruses match hosts’ tRNA pools better than broad-host-range viruses [19]. To adapt to new hosts, after a host shift event, viruses must lower their codon usage bias. Phylogenetic analysis found that distantly related iflaviruses can infect the same host species [49]. This means that host shift events are universal in the *Iflaviridae* family and may be a possible explanation for the phenomenon that the patterns of codon usage in the *Iflaviridae* family do not vary with their host taxa. In summary, the present investigation did not verify our assumption that diverse iflaviruses should possess diverse codon usage patterns. In fact, the overall codon usage in the *Iflaviridae* family was found to be relatively uniform.

## Figures and Tables

**Table 1:** Iflaviruses sharing most of the preferred codons independently of their host taxa, indicating that the preferred codons are not host taxa-specific.

Preferred Codons (aa)	Arachnida	Diptera	Hemiptera	Hymenoptera	Lepidoptera	Non-Insects
GCT(Ala)	✓	✓	✓	✓	✓	✓
AGA(Arg)	✓	✓	✓	✓	✓	✓
CGT(Arg)	✓	✓	✓	✓	✓	✓
AAT(Asn)	✓	✓	✓	✓	✓	✓
GAT(Asp)	✓	✓	✓	✓	✓	✓
TGT(Cys)	✓	✓	✓	✓	✓	✓
CAA(Gln)	✓	✓	✓	✓	✓	✓
GAA(Glu)	✓	✓	✓	✓	✓	✓
GGA(Gly)	✗	✓	✗	✗	✗	✗
GGT(Gly)	✓	✗	✓	✓	✓	✓
CAT(His)	✓	✓	✓	✓	✓	✓
ATT(Ile)	✓	✓	✓	✓	✓	✓
CTT(Leu)	✗	✓	✗	✗	✗	✗
TTA(Leu)	✓	✗	✓	✓	✓	✓
TTG(Leu)	✓	✓	✓	✓	✓	✓
AAA(Lys)	✓	✓	✓	✓	✓	✗
TTT(Phe)	✓	✓	✓	✓	✓	✓
CCT(Pro)	✓	✓	✓	✓	✓	✓
AGT(Ser)	✓	✗	✓	✓	✓	✓
TCA(Ser)	✗	✓	✗	✗	✗	✗
TCT(Ser)	✓	✓	✓	✓	✓	✓
ACT(Thr)	✓	✓	✓	✓	✓	✓
UAT(Tyr)	✓	✓	✓	✓	✓	✓
GTT(Val)	✓	✓	✓	✓	✓	✓

Column titles, Arachnida, Diptera, Hemiptera, Hymenoptera, Lepidoptera, and non-Insects, represent viruses infecting the hosts of Arachnida, Diptera, Hemiptera, Hymenoptera, Lepidoptera, and non-Insects, respectively. A tick symbol (✓) and a cross symbol (✗) represent the host-taxa-groups selecting or not selecting the corresponding codons as the preferred codons, respectively.

## Supplementary Materials

The following are available online at https://www.mdpi.com/1999-4915/11/12/1087/s1. Table S1: Nucleotide composition and effective number of codons in 44 viral genomes; Table S2: Dinucleotides at the 1st–2nd and 2nd–3rd codon positions in 44 viral genomes; and Table S3: RSCU values and preferred codons in six host-taxa-groups.

## References

[1] Plotkin J.B., Kudla G. (2011). Synonymous but not the same: The causes and consequences of codon bias. Nat. Rev. Genet..

[2] Chaney J.L., Clark P.L. (2015). Roles for Synonymous Codon Usage in Protein Biogenesis. Annu Rev. Biophys..

[3] Supek F. (2016). The Code of Silence: Widespread Associations Between Synonymous Codon Biases and Gene Function. J. Mol. Evol..

[4] Im E.-H., Choi S.S. (2017). Synonymous Codon Usage Controls Various Molecular Aspects. Genomics Inform..

[5] Mittal P., Brindle J., Stephen J., Plotkin J.B., Kudla G. (2018). Codon usage influences fitness through RNA toxicity. Proc. Natl. Acad. Sci. USA.

[6] Shackelton L.A., Parrish C.R., Holmes E.C. (2006). Evolutionary basis of codon usage and nucleotide composition bias in vertebrate DNA viruses. J. Mol. Evol..

[7] Yao H., Chen M., Tang Z. (2019). Analysis of Synonymous Codon Usage Bias in Flaviviridae Virus. Biomed. Res. Int..

[8] Shi S.-L., Jiang Y.-R., Liu Y.-Q., Xia R.-X., Qin L. (2013). Selective pressure dominates the synonymous codon usage in parvoviridae. Virus Genes.

[9] Valles S.M., Chen Y., Firth A.E., Guérin D.M.A., Hashimoto Y., Herrero S., de Miranda J.R., Ryabov E., Consortium I.R. (2017). ICTV Virus Taxonomy Profile: Iflaviridae. J. Gen. Virol..

[10] Wilfert L., Long G., Leggett H.C., Schmid-Hempel P., Butlin R., Martin S.J., Boots M. (2016). Deformed wing virus is a recent global epidemic in honeybees driven by Varroa mites. Science.

[11] Geng P., Li W., de Miranda J.R., Qian Z., An L., Terenius O. (2017). Studies on the transmission and tissue distribution of Antheraea pernyi iflavirus in the Chinese oak silkmoth Antheraea pernyi. Virology.

[12] Vootla S.K., Lu X.M., Kari N., Gadwala M., Lu Q. (2013). Rapid detection of infectious flacherie virus of the silkworm, Bombyx mori, using RT-PCR and nested PCR. J. Insect Sci..

[13] Carballo A., Murillo R., Jakubowska A., Herrero S., Williams T., Caballero P. (2017). Co-infection with iflaviruses influences the insecticidal properties of Spodoptera exigua multiple nucleopolyhedrovirus occlusion bodies: Implications for the production and biosecurity of baculovirus insecticides. PLoS ONE.

[14] Carrillo-Tripp J., Bonning B.C., Miller W.A. (2015). Challenges associated with research on RNA viruses of insects. Curr. Opin. Insect. Sci..

[15] Yuan H., Xu P., Yang X., Graham R.I., Wilson K., Wu K. (2017). Characterization of a novel member of genus Iflavirus in Helicoverpa armigera. J. Invertebr. Pathol..

[16] Suzuki T., Takeshima Y., Mikamoto T., Saeki J.D., Kato T., Park E.Y., Kawagishi H., Dohra H. (2015). Genome Sequence of a Novel Iflavirus from mRNA Sequencing of the Pupa of Bombyx mori Inoculated with Cordyceps militaris. Genome Announc.

[17] Silva L.A., Ardisson-Araujo D.M., Tinoco R.S., Fernandes O.A., Melo F.L., Ribeiro B.M. (2015). Complete genome sequence and structural characterization of a novel iflavirus isolated from Opsiphanes invirae (Lepidoptera: Nymphalidae). J. Invertebr. Pathol..

[18] Martin S.J., Brettell L.E. (2019). Deformed Wing Virus in Honeybees and Other Insects. Annu. Rev. Virol..

[19] Tian L., Shen X., Murphy R.W., Shen Y. (2018). The adaptation of codon usage of +ssRNA viruses to their hosts. Infect. Genet. Evol..

[20] O’Leary N.A., Wright M.W., Brister J.R., Ciufo S., Haddad D., McVeigh R., Rajput B., Robbertse B., Smith-White B., Ako-Adjei D. (2016). Reference sequence (RefSeq) database at NCBI: Current status, taxonomic expansion, and functional annotation. Nucleic Acids Res..

[21] Simmonds P., Adams M.J., Benkő M., Breitbart M., Brister J.R., Carstens E.B., Davison A.J., Delwart E., Gorbalenya A.E., Harrach B. (2017). Virus taxonomy in the age of metagenomics. Nat. Rev. Microbiol..

[22] Karlin S., Burge C. (1995). Dinucleotide relative abundance extremes: A genomic signature. Trends Genet..

[23] Sharp P.M., Tuohy T.M.F., Mosurski K.R. (1986). Codon usage in yeast: Cluster analysis clearly differentiates highly and lowly expressed genes. Nucleic Acids Res..

[24] Shi S.L., Jiang Y.R., Yang R.S., Wang Y., Qin L. (2016). Codon usage in Alphabaculovirus and Betabaculovirus hosted by the same insect species is weak, selection dominated and exhibits no more similar patterns than expected. Infect. Genet. Evol.

[25] Zhou J.H., Zhang J., Sun D.J., Ma Q., Chen H.T., Ma L.N., Ding Y.Z., Liu Y.S. (2013). The distribution of synonymous codon choice in the translation initiation region of dengue virus. PLoS ONE.

[26] Wright F. (1990). The ‘effective number of codons’ used in a gene. Gene.

[27] Roychoudhury S., Pan A., Mukherjee D. (2011). Genus specific evolution of codon usage and nucleotide compositional traits of poxviruses. Virus Genes.

[28] Chen Y., Shi Y., Deng H., Gu T., Xu J., Ou J., Jiang Z., Jiao Y., Zou T., Wang C. (2014). Characterization of the porcine epidemic diarrhea virus codon usage bias. Infect. Genet. Evol..

[29] Sueoka N. (1988). Directional mutation pressure and neutral molecular evolution. Proc. Natl. Acad. Sci. USA.

[30] Sueoka N. (1995). Intrastrand parity rules of DNA base composition and usage biases of synonymous codons. J. Mol. Evol..

[31] Sueoka N. (1999). Translation-coupled violation of Parity Rule 2 in human genes is not the cause of heterogeneity of the DNA G+C content of third codon position. Gene.

[32] Suzuki H., Brown C.J., Forney L.J., Top E.M. (2008). Comparison of correspondence analysis methods for synonymous codon usage in bacteria. DNA Res..

[33] Deng W., Wang Y., Liu Z., Cheng H., Xue Y. (2014). HemI: A Toolkit for Illustrating Heatmaps. PLoS ONE.

[34] Hanson G., Coller J. (2017). Codon optimality, bias and usage in translation and mRNA decay. Nat. Rev. Mol. Cell. Biol..

[35] Biswas K.K., Palchoudhury S., Chakraborty P., Bhattacharyya U.K., Ghosh D.K., Debnath P., Ramadugu C., Keremane M.L., Khetarpal R.K., Lee R.F. (2019). Codon Usage Bias Analysis of Citrus tristeza virus: Higher Codon Adaptation to Citrus reticulata Host. Viruses.

[36] Karniychuk U.U. (2016). Analysis of the synonymous codon usage bias in recently emerged enterovirus D68 strains. Virus Res..

[37] Kumar N., Kulkarni D.D., Lee B., Kaushik R., Bhatia S., Sood R., Pateriya A.K., Bhat S., Singh V.P. (2018). Evolution of Codon Usage Bias in Henipaviruses Is Governed by Natural Selection and Is Host-Specific. Viruses.

[38] Khandia R., Singhal S., Kumar U., Ansari A., Tiwari R., Dhama K., Das J., Munjal A., Singh R.K. (2019). Analysis of Nipah Virus Codon Usage and Adaptation to Hosts. Front. Microbiol.

[39] Cristina J., Moreno P., Moratorio G., Musto H. (2015). Genome-wide analysis of codon usage bias in Ebolavirus. Virus Res..

[40] Cristina J., Fajardo A., Sonora M., Moratorio G., Musto H. (2016). A detailed comparative analysis of codon usage bias in Zika virus. Virus Res..

[41] Kliman R.M., Bernal C.A. (2005). Unusual usage of AGG and TTG codons in humans and their viruses. Gene.

[42] Palidwor G.A., Perkins T.J., Xia X. (2010). A General Model of Codon Bias Due to GC Mutational Bias. PLoS ONE.

[43] Svensson E.I.L., Berger D. (2019). The Role of Mutation Bias in Adaptive Evolution. Trends Ecol. Evol..

[44] Zhang W., Zhang L., He W., Zhang X., Wen B., Wang C., Xu Q., Li G., Zhou J., Veit M. (2019). Genetic Evolution and Molecular Selection of the HE Gene of Influenza C Virus. Viruses.

[45] Nasrullah I., Butt A.M., Tahir S., Idrees M., Tong Y. (2015). Genomic analysis of codon usage shows influence of mutation pressure, natural selection, and host features on Marburg virus evolution. BMC Evol. Biol..

[46] Butt A.M., Nasrullah I., Tong Y. (2014). Genome-wide analysis of codon usage and influencing factors in chikungunya viruses. PLoS ONE.

[47] Giallonardo F.D., Schlub T.E., Shi M., Holmes E.C. (2017). Dinucleotide Composition in Animal RNA Viruses Is Shaped More by Virus Family than by Host Species. J. Virol..

[48] Sexton N.R., Ebel G.D. (2019). Effects of Arbovirus Multi-Host Life Cycles on Dinucleotide and Codon Usage Patterns. Viruses.

[49] Liu S., Chen Y., Bonning B.C. (2015). RNA virus discovery in insects. Curr. Opin. Insect Sci..

